# From conservation scores to language models: a feature engineering perspective on machine learning for genetic variant interpretation

**DOI:** 10.1093/bib/bbag552

**Published:** 2026-10-11

**Authors:** Matteo Bulloni, Martina Vescio, Federico De Mori Bajolin, Linda Pattini

**Affiliations:** Department of Electronics, Information and Bioengineering, Politecnico di Milano, Piazza Leonardo da Vinci 32, 20133 Milan, Italy; Cardio-Tech Lab, Centro Cardiologico Monzino IRCCS, via Carlo Parea, 4, 20138 Milan, Italy; Department of Electronics, Information and Bioengineering, Politecnico di Milano, Piazza Leonardo da Vinci 32, 20133 Milan, Italy; Department of Electronics, Information and Bioengineering, Politecnico di Milano, Piazza Leonardo da Vinci 32, 20133 Milan, Italy; Cardio-Tech Lab, Centro Cardiologico Monzino IRCCS, via Carlo Parea, 4, 20138 Milan, Italy

**Keywords:** genetic variant effect prediction, evolutionary conservation, feature engineering, genomic language models, protein language models, deep learning

## Abstract

The interpretation of genetic variants remains central to precision medicine, and machine-learning-based variant effect prediction tools have become indispensable for supporting decision-making processes. In this review we trace the progression from hand-crafted features and traditional machine learning classifiers, through supervised ensembles, to deep-learning architectures and foundation models that learn representations directly from biological sequences. We classify methods for genetic variant effect prediction by underlying machine learning model and variant type handled, from missense to non-coding and structural, as well as across feature categories, describing their biological rationale, adoption rate, and known limitations. We further examine three cross-cutting methodological challenges—feature orthogonality, data circularity, and training data bias—that affect reliability and clinical applicability, providing a critical overview of the current landscape and the challenges that lie ahead.

## Introduction

Over the last two decades, technologies made it possible to generate an unprecedented amount of data regarding the genetic variability present in the human population [1–3].

As the number of identified genetic variants increases, so do the challenges in interpreting them, making it difficult to effectively use this information in clinical patient management [4, 5].

The first algorithms developed to predict the functional consequences of genetic variants—variant effect prediction (VEP)—produced a score mainly based on amino acids substitutions and evolutionary conservation. For example, the Grantham score focused only on physicochemical similarity between amino acids, assigning a lower score to substitutions between amino acids with similar characteristics [6]. Subsequent algorithms, such as SIFT and GERP++, generated predictions based on sequence conservation, penalizing mutations at evolutionary conserved sites [7, 8]. In more recent years, multiple machine learning algorithms have been developed to tackle the problem of VEP, as they allow considering multiple variant-associated features at the same time to increase the accuracy of the prediction, as well as modeling non-linear relationships between features and variant pathogenicity. The employment of such methods has in the past decade led to steady improvements in the interpretation of the functional consequences of variants [9, 10].

Several recent reviews have examined these approaches from complementary perspectives. Riccio et al. [11] cataloged over 100 VEP tools and introduced the VEP Finder resource. Garcia et al. [12] categorized tools by methodology before adoption of protein and genomic language models. Wang et al. [13] reviewed deep learning approaches for non-coding variant prediction, while Gerasimavicius et al. [14] summarized the growing role of protein structural information, particularly AlphaFold-derived features, in VEP. Recent benchmarking studies have also advanced the field: Livesey and Marsh established rigorous benchmarking standards and highlighted data circularity issues [15, 16]; Radjasandirane et al. [17] evaluated 65 VEPs across variants of differing prediction difficulty; and Notin et al. [18] introduced the ProteinGym benchmark for systematic evaluation against deep mutational scanning and clinical datasets.

In this review, we trace the methodological evolution of VEP tools, presenting a comprehensive account of the various categories of features employed by machine-learning-based (ML-based) algorithms and their evolution over the past decade (Fig. 1), and highlighting the progressive shift, still in occurrence, from traditional machine learning approaches based on hand-engineered features to deep learning-based (DL-based) models.

**Figure 1:**
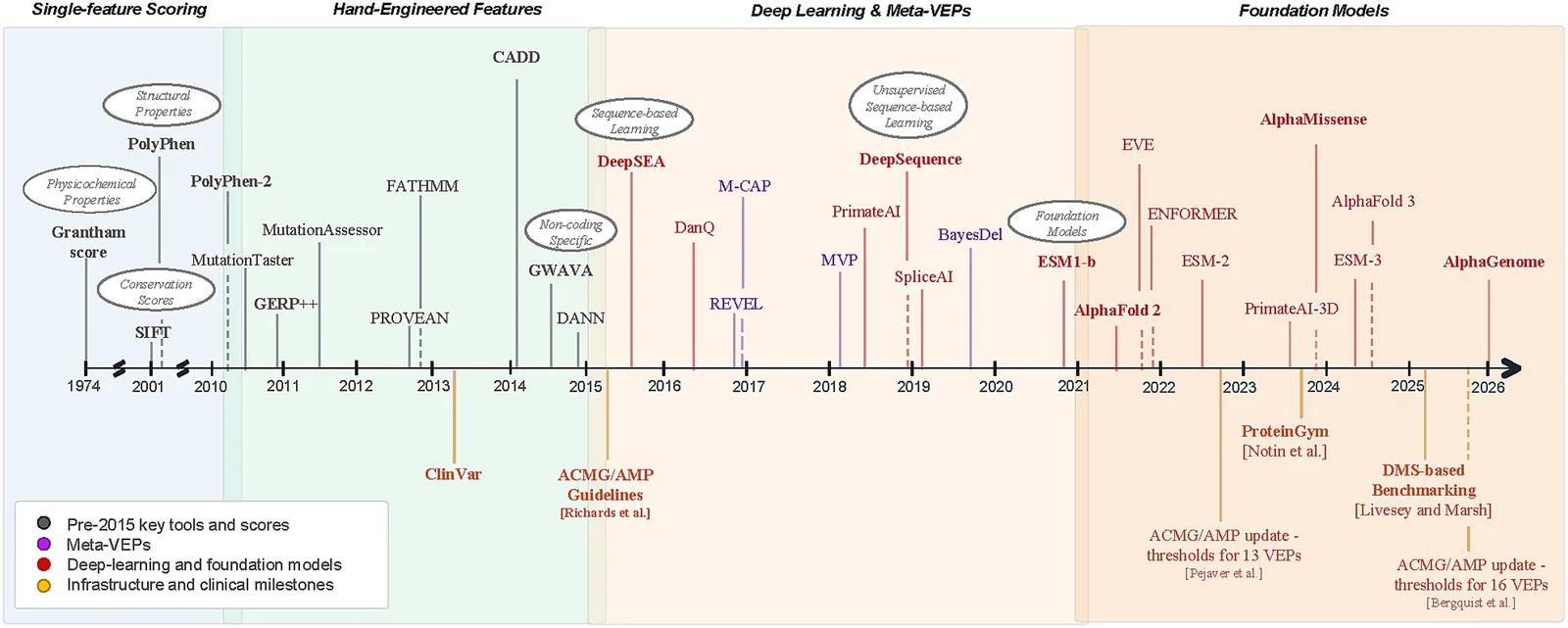
Timeline of variant effect prediction tools and milestones (1974–2026). The figure traces the evolution of the VEP field across four methodological eras: single-feature scoring, hand-engineered feature integration, meta-VEPs, deep learning and foundation models. Entries are color-coded by category: pre-2015 key tools and scores (grey), meta-VEPs (purple), deep-learning and foundation models (red). The bottom part of the timeline includes some key milestones of the field, in orange. Ovals denote conceptual innovations.

Additionally, we attempt to present and underscore key issues affecting the field of VEP, such as that of data circularity feature orthogonality, data circularity, and training data biases.

### ML-based variant effect prediction

Machine learning-based VEP tools distinguish pathogenic from benign genetic variants by learning patterns from biological features. The choice and representation of these features fundamentally determines what biological signals a model can capture and, consequently, its predictive accuracy and generalizability. This feature selection process involves key trade-offs and challenges along two fundamental axes: interpretability and orthogonality. Hand-engineered features (such as conservation scores or physicochemical properties) offer biological interpretability but may fail to capture complex, non-linear relationships. Learned representations from deep neural networks, instead, can model these relationships, but function as black boxes where the basis for predictions is opaque. Recent ‘zero-shot’ methods and the underlying foundation models—predicting variant effects without training on pathogenicity labels, directly deriving signal from evolutionary or structural patterns—offer an intermediate approach. As the field moves away from white-box approaches, interpretability challenges become central to clinical adoption. A similar tension exists between using highly correlated features that capture overlapping signal (potentially overfitting to shared biases) and selecting diverse features that provide independent information. For example, conservation-based features assume that evolutionary constraint predicts human disease relevance—an assumption that fails for late-onset conditions and recent human adaptations, whereas protein structure features fail in disordered regions lacking stable conformations.

The standards set by the American College of Medical Genetics and Genomics and the Association for Molecular Pathology (ACMG/AMP) [19] provide a formal framework for rare variant interpretation, assigning specific codes to the different types of evidence which can be employed to classify a variant. Within the ACMG/AMP framework, computational predictions are indeed recognized as supporting evidence for variant pathogenicity or benignity, but only when tools reach accuracy thresholds that however remain inconsistently defined across clinical laboratories. While recent recommendations by the ClinGen Sequence Variant Interpretation Working Group—established in 2013 and responsible for updating and operationalizing the ACMG/AMP guidelines—have proposed statistically calibrated thresholds for a subset of 16 existing VEP tools [20, 21], for the vast majority of methods no such standardized conversion between raw prediction scores and clinical evidence strength exists. As a consequence, the gap between research performance and clinical validation is a recurring theme across feature categories: SIFT [7], PolyPhen-2 [22] and CADD [23] remain the most widely cited tools in clinical variant interpretation, despite the emergence of tools with superior benchmark performance, reflecting the inertia of established workflows and the high barrier for validating new ‘black box’ models and features.

The development of machine-learning-based VEP tools has historically been defined by the availability of training data, as ML-based models are generally very data-hungry. The release of the ClinVar database in 2013 [24] provided a centralized, public archive of reports on the relationships between human variants and phenotypes, which has since progressively grown in both size and recognition, becoming the standard ground truth for the field. This marked a substantial shift specifically for ML-based VEP tools trained on clinical pathogenicity labels: tools developed after 2013 increasingly utilized ClinVar for training, enabling learning on a massive scale but simultaneously introducing systematic biases and data circularity issues. Not all ML-based VEP tools are equally affected—those relying on evolutionary proxies, functional genomic annotations, or self-supervised sequence modeling do not train on pathogenicity labels and are therefore not directly implicated. For tools that do, however, the biases introduced include the over-representation of severe, early-onset Mendelian diseases and European ancestry populations, reflecting the composition of ClinVar itself. Data circularity issues, instead, progressively emerged as an increasing number of tools were trained and evaluated on overlapping subsets of the same clinical databases, and as variant classifications within those databases were themselves informed by earlier computational predictions—creating feedback loops where predictors indirectly contribute to the labels against which they are assessed. These issues represent a persistent and compounding problem for VEP tools, which must be taken into account and addressed to ensure reliable future developments of the field (Fig. 2).

**Figure 2:**
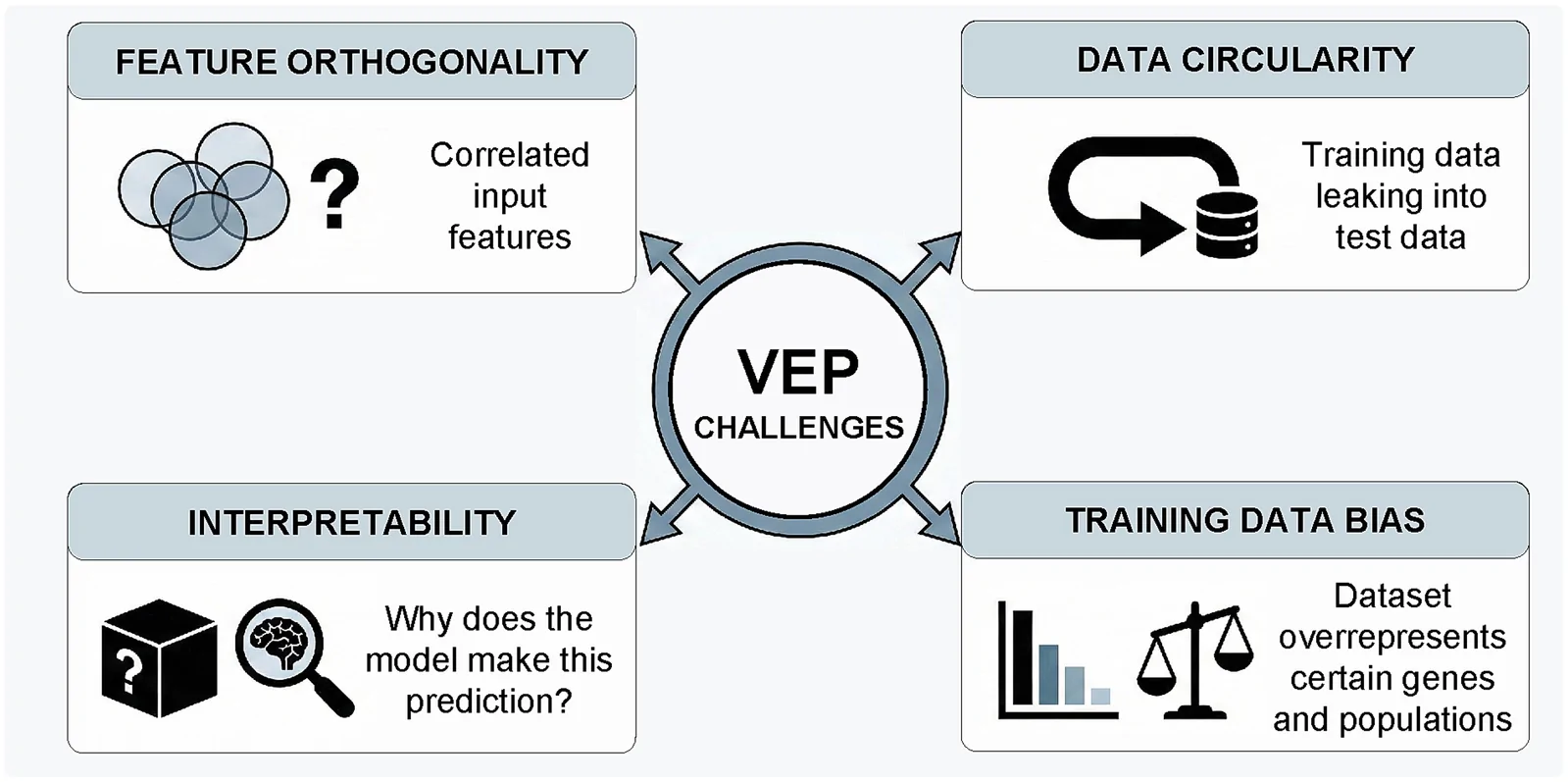
Key methodological challenges in variant effect prediction. Four cross-cutting issues affect VEP reliability: feature orthogonality (correlated input features capturing redundant signal), data circularity (training data leaking into evaluation benchmarks), training data bias (over-representation of certain genes, populations, and variant types), and interpretability (difficulty explaining model predictions).

Features employed by VEP tools nowadays fall into several broad categories: evolutionary conservation, amino acid physicochemistry, protein structure, regulatory and epigenomic annotations, population genetics, and—increasingly—learned representations from language models. The following sections describe each feature category in detail. Among the tools included in this review (a complete catalogue is provided in Supplementary Table 1), referring to the last decade, conservation-based features are most prevalent (used by 64%), followed by protein structure features (31%) and sequence context (31%). Features derived from protein and genome language models, grouped together under the ‘learned representations’ term, also constitute a recent but rapidly growing category (35%), the culmination of the progressive shift in place towards deep-learning-based models (Table 1).

**Table 1:** Feature categories employed by the 174 VEP tools reviewed in this study.

Feature Category	Count (%)	
Conservation-based	112 (64.4%)
Learned representations	61 (35.1%)
Protein structure	54 (31.0%)
Sequence context	53 (30.5%)
Physicochemical properties	50 (28.7%)
Pre-2015 pathogenicity-label-trained VEP scores	46 (26.4%)
Population frequency	44 (25.3%)
Gene/domain constraint	34 (19.5%)
Epigenomic	32 (18.4%)
Post-2014 pathogenicity-label-trained VEP scores	31 (17.8%)
Splicing	29 (16.7%)
TF binding	23 (13.2%)
Regulatory elements	17 (9.8%)
Network/pathway	17 (9.8%)
Codon usage bias	5 (2.9%)

### Features for coding variants

The features discussed in this section exploit the biological priors relevant to this context, ranging from evolutionary conservation and amino acid physicochemistry to 3D structural context, gene-level constraint, population frequency, biological networks, and codon usage. Of these, conservation scores and population frequency hold for non-coding regions as well; they are discussed here given their historical roots in protein-coding sequence analysis and their foundational role in the coding VEP tools that initiated the field [7, 22, 25, 26].

### Conservation-based features

Evolutionary conservation remains the most widely used feature category based on the fundamental premise that genomic positions preserved by evolution across species are more likely to be functionally important, and variants at these positions are more likely to be pathogenic. Several complementary approaches that quantify this expected tendency have been developed throughout the years. PhyloP measures nucleotide-level conservation by computing the rate of base substitution at each genomic position and comparing it to a neutral model of evolution [27]. Positive phyloP scores indicate positions evolving slower than expected (conservation), while negative scores indicate accelerated evolution. PhastCons models conservation as a hidden Markov process—a probabilistic model that infers underlying conservation states from observed sequence patterns—identifying contiguous conserved elements rather than individual nucleotides [28]. This approach captures the fact that functional regions typically span multiple bases. GERP++ (Genomic Evolutionary Rate Profiling) estimates ‘rejected substitutions’—mutations that would have occurred under neutral evolution but were eliminated by selection. This metric directly quantifies selective pressure at each position [8]. SiPhy, instead, analyses substitution patterns rather than just rates, detecting constraint even when the substitution rate appears normal but the pattern of changes is biased [29]. More recently, tools have started to incorporate more expanded and heterogeneous conservation data. CADD v1.7, e.g. integrates scores from the Zoonomia project’s alignment of 241 mammalian genomes, which led to an improvement in correlation with minor allele frequency reflecting increased power from denser phylogenetic sampling [30]. Beyond single-position scores, ultra-conserved elements (UCEs) highlight highly constrained genomic regions beyond single-position scores. At the protein level, conservation is captured by position-specific scoring matrices (PSSMs), which reflect amino acid variation across homologs. Coevolution analysis further identifies residue pairs that evolve together, suggesting functional or structural coupling [31].

### Protein sequence and physicochemical properties

When a missense variant alters the amino acid sequence, the physicochemical differences between wild-type and mutant residues provide important predictive signal. Hydrophobicity scales, such as the Kyte-Doolittle index, quantify the tendency of amino acids to be buried in the protein interior versus exposed to solvent [32]. Large changes in hydrophobicity upon mutation tend to disrupt protein folding by destabilizing the hydrophobic core. Similarly, changes in polarity, charge and side chain volume perturb electrostatic interactions, hydrogen bonding networks, and steric packing. Substitution matrices provide an evolutionary perspective on amino acid exchangeability. The BLOSUM matrix, derived from alignments of related protein sequences, assigns scores to each possible substitution based on how frequently it is observed in nature [33]. The Grantham distance incorporates composition, polarity, and molecular volume differences between amino acids [6]. More detailed biochemical properties include molecular weight, isoelectric point, and side chain accessible surface area. Statistical potentials derived from known protein structures, such as the Skolnick potential for sequence neighbours, capture energetic consequences of mutations within their local context [34].

### Protein structure features

Protein 3D structure is key to interpreting variant effects: mutations in the hydrophobic core tend to destabilize proteins, whereas surface changes are often tolerated. Structure-based tools therefore use features like solvent accessibility, secondary structure, residue burial, and proximity to functional sites (e.g. active or binding sites). They may also estimate changes in folding stability (ΔΔG).

A major limitation is that these methods perform poorly in intrinsically disordered regions (IDRs), which lack stable structure—despite comprising ~25% of residues and being present in 58% of human proteins [35]. Additionally, variant distributions differ dramatically between ordered and disordered regions. Only 3.6% of ClinVar pathogenic missense variants occur in IDRs, compared to 39.8% of benign variants—an 11-fold enrichment of benign variants in disordered regions [35]. This skewed distribution creates a systematic bias: VEPs trained on datasets where most pathogenic variants occur in structured regions will underperform on the ~1550 pathogenic missense variants in disordered regions identified in ClinVar as of 2024 [35]. Classical tools including SIFT and PolyPhen-2 show greater than 10% sensitivity reduction for IDR variants. IDR pathogenic variants operate through distinct mechanisms poorly captured by structure-based features: disruption of short linear motifs, alteration of post-translational modification sites, and perturbation of liquid–liquid phase separation behaviour. These mechanisms require specialized feature engineering beyond traditional structural descriptors, a jump that was first adequately addressed by deep-learning approaches. This shift was marked by AlphaFold2, whose methodology was published and open-sourced in 2021, representing a landmark achievement in protein structure prediction [36]. Crucially for VEP, AlphaFold provides a per-residue confidence metric (pLDDT, ranging from 0–100) that effectively flags IDRs: scores below 70 indicate probable disorder, enabling systematic identification of regions where structure-based features are unreliable. Beyond disorder prediction, AlphaFold’s predicted structures can serve as input features for VEPs that incorporate structural context. This application of deep learning architectures—including AlphaFold itself—to directly predict pathogenicity scores represents, however, a distinct paradigm that will be discussed later.

### Codon usage bias

For coding variants, synonymous mutations can affect protein function through codon optimality effects on translation speed and co-translational folding. Rare codons slow ribosome transit, potentially affecting folding kinetics for nascent polypeptides. Different synonymous codons are recognized by tRNAs at different abundances, creating a ‘codon bias’ that varies across genes and organisms. synVep, e.g. incorporates multiple codon bias metrics—including the Codon Adaptation Index, tRNA Adaptation Index, and a novel codon autocorrelation measure—as features for predicting synonymous variant effects [37]. While niche compared to conservation and structure features, codon usage ones capture a distinct biological mechanism—one particularly relevant as evidence accumulates that synonymous variants are not uniformly neutral. These features complement the traditional focus on amino acid-changing variants.

### Gene- and Domain-level resolution metrics

Beyond position-specific features, gene-level metrics capture the overall tolerance of a gene to functional variation within the human population. Multiple indices quantify different facets of this evolutionary constraint. The Residual Variation Intolerance Score (RVIS) compares observed functional variants in a gene to the number expected based on gene size and total variant count; genes with fewer functional variants than expected are classified as intolerant [38]. Similarly, the Gene Damage Index (GDI) measures accumulated mutational damage in healthy populations [39]. The pLI score estimates whether a gene is intolerant to heterozygous loss-of-function variants, indicating haploinsufficiency [25]. The LOEUF score provides a more refined measure of the same tolerance, aided by confidence intervals [40].

The Domain Damage Index (DDI) quantifies depletion of rare missense variants within protein domains, operating independently of gene-level constraint scores. Importantly, DDI correlates only moderately with CADD (Spearman ρ = 0.443), indicating substantial orthogonal information [41].

A critical limitation of gene- and protein domain-level metrics is calibration heterogeneity. Genome-wide calibration of VEP scores, while practical for establishing ACMG/AMP evidence thresholds, masks substantial gene-specific variation. An analysis of 3668 disease-relevant genes found that ~70% of evaluable gene-specific score intervals showed ‘trending discordant’ performance relative to genome-wide calibrations [42].

### Population frequency features

Allele frequency in human populations provides strong evidence about variant pathogenicity. Modern tools integrate allele frequencies from large databases including gnomAD [40], which supersedes the earlier ExAC [25], and the 1000 Genomes Project [43]. Frequencies are typically stratified by population (African, European, East Asian, etc.) to account for population-specific variants. The maximum allele frequency across populations is frequently used as a filtering criterion. Some tools explicitly exclude allele frequency, or are trained specifically to tackle rare variants via tailored training or methodological setups [44, 45]. REVEL represents one of the most well-recognized such examples, designed to perform well on rare variants by training on rare neutral variants rather than common polymorphisms [44].

### Network and pathway features

Some tools incorporate biological network information beyond individual protein properties. The premise is that proteins central to disease-relevant pathways may be more sensitive to perturbation—variants in ‘hub’ proteins with many interaction partners can have outsized phenotypic effects. DEOGEN integrates protein–protein interaction (PPI) network degree from ConsensusPathDB alongside pathway annotations [46]. DVA transforms STRING database interactions via node2vec graph embeddings, learning topological features that predict variant impact [47]. Ozturk and Carter compute extensive network centrality measures including degree, betweenness, closeness and eigenvector centrality at both protein and residue levels [48]. These approaches bridge variant-level prediction with systems biology, recognizing that a variant’s functional impact depends on the protein’s position in cellular networks.

### Features for non-coding variants

While coding variants alter protein sequence directly, the vast majority of the genome is non-coding and requires fundamentally different features for effect prediction, able to capture the regulatory landscape even across cell types and tissues.

### Epigenomic features

Chromatin accessibility, measured by DNase-seq or ATAC-seq, identifies regions of open chromatin where DNA is accessible to transcription factors and regulatory machinery. Variants in accessible chromatin regions are more likely to have functional effects than those in closed, inaccessible regions. The ENCODE [49] and Roadmap Epigenomics [50] projects generated chromatin accessibility profiles across hundreds of cell types, enabling tissue-specific effect prediction. A key element to establish accessibility are histone modifications, marking distinct chromatin states associated with different regulatory functions: H3K4me3 marks active promoters; H3K4me1 and H3K27ac mark active enhancers; H3K27me3 marks Polycomb-repressed regions; H3K9me3 marks constitutive heterochromatin. By integrating multiple histone marks, tools classify genomic regions into functional chromatin states. Three-dimensional genome organization provides an additional layer of regulatory context: variants disrupting topologically associating domains (TAD) boundaries or CTCF (CCCTC-binding factor) binding sites can cause regulatory rewiring with pathogenic consequences.

Despite their potential and relatively widespread application in non-coding variant effect prediction, a key limitation of epigenomic features is tissue specificity—few tools systematically address tissue matching [51] and temporal dynamics.

### Transcription factor binding features and regulatory element annotations

Transcription factors (TFs) regulate gene expression by binding specific DNA: variants disrupting TF binding sites can alter gene regulation and contribute to disease. Position weight matrices (PWMs) are probability matrices defining sequence preferences at each position of a binding. By scoring how well a sequence matches the PWM before and after mutation, tools can predict whether variants create or disrupt binding sites. The JASPAR [52] and HOCOMOCO [53] databases provide curated PWMs for hundreds of human TFs.

Curated annotations of regulatory elements provide explicit labels for functional regions. Promoters, typically located within few kilobases upstream of transcription start sites, contain binding sites for the basal transcription machinery. Hence, variants disrupting core promoter elements can abolish gene expression. Enhancers, instead, activate transcription from distal locations through chromatin looping, in a tissue-specific manner. Databases such as GeneHancer [54], FANTOM5 [55], and the ENCODE candidate *cis-*regulatory element registry [56] provide genome-wide enhancer annotations. Variants disrupting enhancer activity can alter gene expression through long-range dysregulation of target genes. Untranslated regions (UTRs) at transcript ends contain regulatory elements affecting mRNA stability, localization, and translation efficiency. 3’UTR variants can disrupt polyadenylation signals and microRNA binding sites; 5’UTR variants may affect translation initiation or upstream open reading frames. Expression quantitative trait loci (eQTL) data from projects such as GTEx [57] provide direct evidence that specific variants affect gene expression in particular tissues. While not a feature per se, eQTL associations are used by some tools to validate predictions or as training labels [58].

### Sequence context features

Local sequence context provides predictive signal independent of explicit annotations. For example, GC content correlates with gene density, chromatin state, and recombination rate [59]. CpG dinucleotides are preferentially located in regulatory regions and are hypermutable due to spontaneous deamination of methylated cytosine in thymine. K-mer composition—the frequency of short nucleotide sequences (typically 1–6 bases)—captures local sequence patterns predictive of function. Flanking nucleotide context affects mutation rates and may influence TF binding through cooperative effects. Repetitive elements including SINEs, LINEs (Short/Long Interspersed Nuclear Elements) and satellite repeats are generally depleted of functional elements, helping distinguish variants in unique sequences from those in repetitive DNA [23]. Finally, human-specific constraint metrics complement cross-species conservation. The JARVIS score, for instance, identifies positions under selective constraint within human populations using allele frequency spectrum analysis [60]. Overall, sequence context features capture local patterns without relying on external annotations, providing baseline signal even in poorly annotated genomic regions.

### Splicing features

Splicing features bridge coding and non-coding prediction, as variants in both exons and introns can disrupt transcript processing. Canonical splice sites at exon-intron boundaries follow strong sequence consensus (GT-AG for most introns, GC-AG for a minority, and AT-AC for minor introns). Variants disrupting these dinucleotides almost always abolish splicing, while variants in the extended splice site region (positions −3 to +6 at the donor, −20 to +3 at the acceptor) can reduce splicing efficiency. Here, VEP is carried out via position weight matrices and maximum entropy models, which quantify sequence preferences at donor and acceptor sites. Exonic splicing enhancers (ESEs) and silencers (ESSs) are instead typically characterized as motifs within exons that recruit splicing factors and modulate splice site recognition. Exonic variants can disrupt these elements even when they do not directly affect splice sites, causing exon skipping or aberrant splicing, and various tools incorporate forms of ESE/ESS scores [61, 62]. Another way of quantifying effects on splicing employed by some VEPs is predicting branch points, by identifying the adenosine residue targeted during the first step of splicing, typically located 18–40 nucleotides upstream of the acceptor site [63, 64], as variants disrupting branch points can be cause of disease.

Deep-learning-based splice predictors have substantially improved prediction of non-canonical splicing effects, with multiple tools released throughout the years quantifying this disruption in different ways. SpliceAI predicts the probability that variants create or disrupt splice sites anywhere in the genome, including deep intronic regions where traditional consensus-based approaches fail [65]. MMSplice predicts quantitative changes in splice site usage and exon inclusion levels [66]. ConSpliceML combines regional splicing constraint with per-nucleotide predictions [67].

### Features for insertions, deletions and structural variants

Beyond single nucleotide ones, insertions, deletions, and structural variants represent a substantial portion of pathogenic variants, and require distinct feature engineering approaches. In fact, indels are present in clinical databases such as ClinVar and HGMD [68], yet most VEP tools focus exclusively on Single Nucleotide Variants (SNVs). Small indels (<50 bp) represent the easiest upgrade from SNVs, and are handled by several tools [69]. Frameshift indels that disrupt the reading frame typically cause loss of function, but the mechanism depends on position, and this poses a greater prediction challenge. Frameshifts early in the transcript usually trigger nonsense-mediated decay (NMD), a cellular surveillance pathway that degrades transcripts with premature termination codons located >50–55 nucleotides upstream of the final exon-exon junction. Frameshifts in the last exon, or those creating premature termination codons within the final ~50 nucleotides before the last exon-exon junction, may instead escape NMD and produce truncated proteins with dominant-negative or gain-of-function effects [70]. In-frame indels (preserving the reading frame) may be tolerated or pathogenic depending on the affected protein region; those disrupting functional domains or protein–protein interaction interfaces are more likely pathogenic. Among the different tools targeting indels, PROVEAN scores indel effects using sequence homology, measuring how well the mutant sequence aligns to homologs compared to wild-type [71], while CADD incorporates reading frame analysis and distance to transcript boundaries [30]. FATHMM-indel specifically addresses non-coding indels using conservation and epigenomic features [72]. MutPred-Indel predicts molecular mechanisms of pathogenic indels including NMD susceptibility [73].

Structural variants (SVs) including large deletions, duplications, inversions, and translocations present distinct challenges due to their size (>50 bp to megabases) and complex genomic effects. The ACMG provides distinct guidelines for this category of events. Short tandem repeat expansions, which cause numerous neurological disorders, require specialized features capturing repeat unit composition and expansion thresholds [74]. Tools such as DeepSVP and StrVCTVRE combine dosage sensitivity scores (pLI, haploinsufficiency, and triplosensitivity scores), gene constraint metrics and phenotype ontology matching to prioritize pathogenic SVs [75, 76]. SvAnna integrates SV overlap with regulatory elements, TAD boundaries, and gene annotations for clinical interpretation [77]. All these approaches recognize that SV pathogenicity depends on multiple factors: which genes are affected, whether those genes are dosage-sensitive, whether regulatory elements are disrupted and whether the SV disrupts 3D chromatin organization. Unlike VEP for SNVs and short indels, the development of machine-learning models for structural variant pathogenicity has been constrained by the limited availability of high-quality labelled datasets. The rarity of confidently classified pathogenic SVs, the heterogeneity of SV mechanisms, and the wide variation in variant size and genomic context have favoured models based on engineered genomic annotations over data-driven representation learning.

### Other VEP tools’ prediction scores as features

Beyond primary biological features, many tools incorporate predictions from other VEPs as input features, raising important methodological considerations. Approximately 30% of reviewed tools employ among their input features at least one score from a core VEP that was originally trained on pathogenicity labels itself. This not only technically makes them ‘meta-predictors’ that ensemble multiple approaches, but raises a further issue. The distinction between methodologically sound ensemble methods and problematic meta-VEPs, in fact, is best understood through data circularity: a meta-predictor is only potentially problematic when its component scores were trained on data that overlaps with the ensemble’s training or evaluation sets. This phenomenon, further discussed later, leads to inflated performance estimates due to the model being evaluated on the same data it was trained on. Thus, not all VEP scores carry the same circularity risk. For example, tools that are unsupervised, trained on evolutionary proxies (e.g. CADD, DANN [78]), or relying on functional genomic annotations (e.g. DeepSEA [79], SpliceAI) do not train on clinical pathogenicity labels, and therefore do not propagate label leakage when their scores are used as input features. By contrast, tools trained directly on clinical pathogenicity databases such as ClinVar, HGMD, or UniProt [80] disease annotations create a direct channel for circularity.

To further refine this distinction, we split the meta-VEP category based on the release date of the pathogenicity-label-trained core VEPs whose scores they incorporate. We set an arbitrary boundary at 2015, reflecting the popularization of ClinVar (first released in 2013) as the field’s dominant training and benchmarking resource—a development that substantially increased the likelihood of overlapping label sets across tools developed thereafter. The ‘post-2014’ heuristic used in this review to identify meta-VEPs should be viewed as an additional practical proxy to quickly provide an approximate estimate of the likelihood of data dependence issues. In fact, among pre-2015 tools, PolyPhen-2, MutationTaster [81], FATHMM [82], and MutPred [83] were indeed trained on pathogenicity labels, but on smaller, largely independent datasets that predate the maturation of ClinVar as the field’s dominant training and benchmarking resource. Using predictions from these tools as features in newer models is thus less risky, because the source predictions predate current training data. Post-2014 pathogenicity-label-trained meta-VEPs, instead, including REVEL [44], M-CAP [84], MVP [85] and DEOGEN2 [86] emerged alongside the growth of ClinVar and other clinical databases that now form standard training and evaluation sets. Additionally, throughout the years more VEP tools were developed with the explicit aim of incorporating the largest possible number of individual VEP scores, exacerbating the data overlap issue: MetaRNN, e.g. ensembles 16 VEP scores including REVEL and M-CAP [87]; DTreePred integrates over 20 classical and modern predictors [88]. In principle, these meta-VEPs achieve strong benchmark performance, as they leverage complementary strengths of component predictors. However, test set variants for these meta-VEPs may have been included in their component tools’ training set, inflating the apparent ensemble performance. Even more subtly, clinical databases like ClinVar may have classified variants partly using those component tools in the first place, creating circularity where the evaluation ‘ground truth’ is not independent of the predictor. These different kinds of data leakage issues are also further discussed and formally categorized in the Methodological Considerations section later in the paper.

Due to these issues, several recent tools explicitly avoid using scores from pathogenicity-label-trained VEPs, instead learning from biological sequences, protein structures, and population genetics [45, 89, 90]. These ‘core VEPs’ provide independent predictions that can be safely combined with other tools or used when component independence is required for statistical validity. In summary, meta-VEPs offer practical performance gains but require careful validation on held-out datasets and should not be used when the component scores informed variant classifications in the evaluation set.

### Deep-learning-based VEP

The deep learning revolution has transformed variant effect prediction by fundamentally changing how biological features are represented. While traditional VEPs rely on hand-crafted features—conservation scores computed from alignments, physicochemical properties from substitution matrices, structural annotations from databases—deep-learning-based approaches learn predictive representations directly from raw sequence data (Fig. 3). This shift does not mean that conservation, structure, or physicochemistry cease to matter; rather, these biological signals are encoded within the evolutionary and biophysical constraints of protein and genomic sequences themselves. Deep neural networks trained on millions of sequences learn to extract these implicit patterns without explicit feature engineering, effectively discovering latent representations that capture the same underlying biology through a fundamentally different computational paradigm. It is important to note that the foundation models discussed in this section—Genomic Language Models (GLMs) and Protein Language Models (PLMs)—do not directly output pathogenicity scores. Instead, they produce embeddings, attention weights or likelihood scores that capture sequence context. Converting these outputs into variant effect predictions requires additional steps: computing log-likelihood ratios between wild-type and mutant sequences (zero-shot prediction), fine-tuning on labeled variant data, or using the embeddings as input features for downstream classifiers. The tools discussed below span this spectrum of approaches, from zero-shot methods like ESM-1v [91] to supervised fine-tuned models and meta-predictors that integrate language model embeddings with other features.

**Figure 3:**
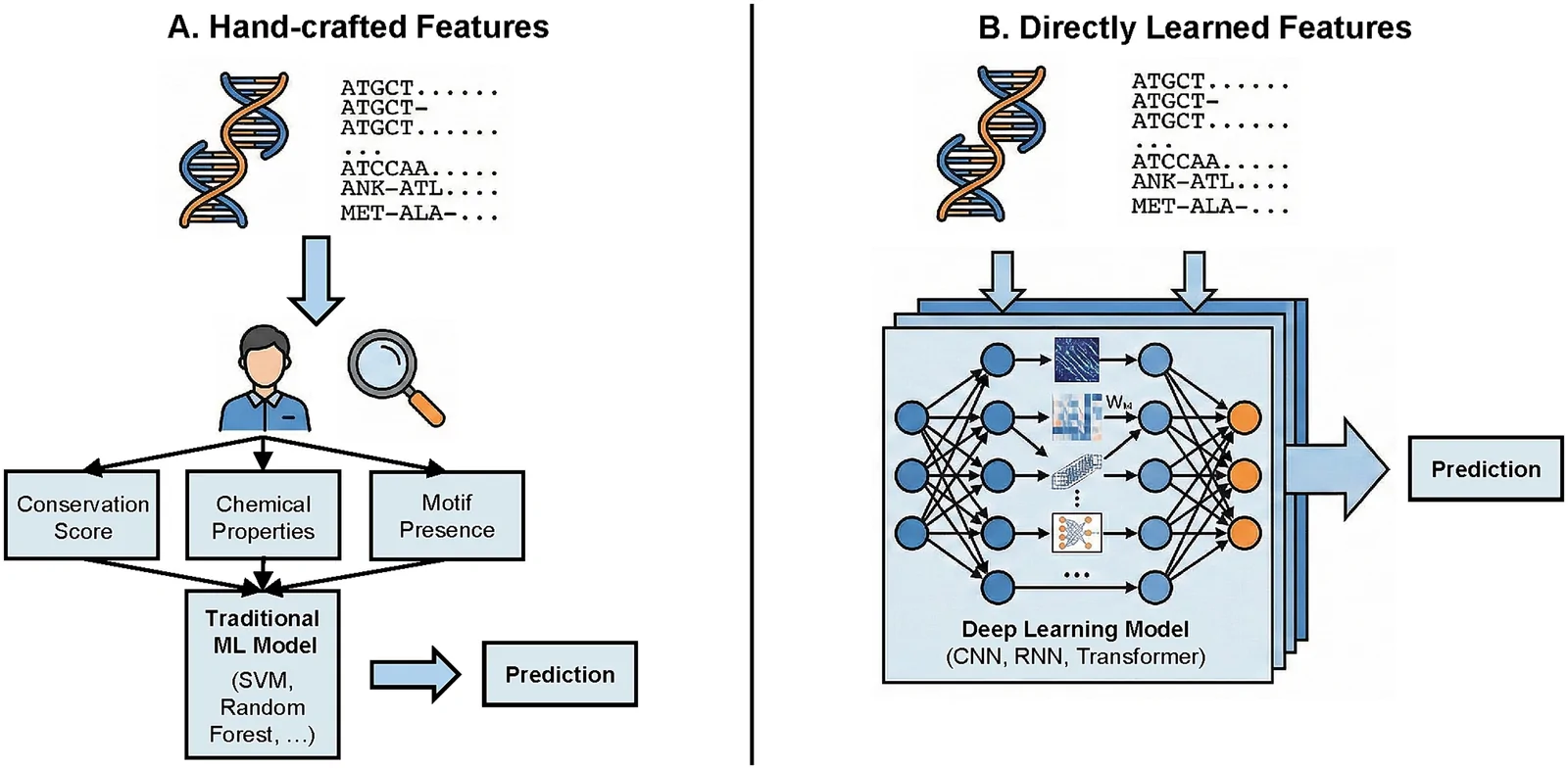
Comparison of hand-crafted and directly learned feature paradigms in variant effect prediction. (A) In the traditional approach, domain experts derive interpretable features—such as conservation scores, physicochemical properties, and motif annotations—which are fed into conventional machine learning models. (B) In the deep-learning paradigm, raw sequence data are provided directly to deep neural networks, which learn predictive representations end-to-end without explicit feature engineering.

### Genomic language models

A transformative development in VEP has been training deep neural networks to learn predictive representations directly from DNA sequence. These models are grouped under the name Genomic Language Models (GLMs), and their core input feature is the genomic sequence itself, typically one-hot encoded (a binary matrix where each row represents a nucleotide position with four columns for A, C, G, T). DeepSEA pioneered this approach in 2015, training a deep CNN on 1-kilobase DNA sequences to predict 919 chromatin features including TF binding, DNase hypersensitivity, and histone modifications [79]. Crucially, models like these learn to predict chromatin state from sequence alone—they do not use chromatin data as input features, but rather as training labels: by comparing predictions for reference and alternative alleles, the model scores variant effects on predicted chromatin state. During this training, the network learns relevant sequence motifs and their combinatorial interactions. Subsequent GLMs expanded both scope and accuracy compared to DeepSEA, each following its own specific path. Sei predicts over 21,000 chromatin profiles from 4-kilobase sequences and groups these profiles into a ‘sequence class’ vocabulary of 40 regulatory activity types. These classes are then further clustered into eight functional categories: promoter, enhancer, CTCF, TF-binding, transcription, polycomb-repressed, heterochromatin, and low-signal [92]. Enformer extended the receptive field to over 100 kilobases using a transformer architecture with self-attention (a mechanism allowing the model to weigh the importance of different sequence positions relative to each other), enabling the model to capture long-range regulatory interactions including enhancer-promoter communication within typical TAD boundaries [93]. The most recent genomic foundation models, however, have come to process megabase-scale contexts. AlphaGenome uses a hybrid architecture combining convolutional layers (for local DNA patterns) with transformer modules (for long-range dependencies) to accept input windows of up to 1 million base pairs [94]. This expanded input context—orders of magnitude larger than early CNNs—captures entire regulatory domains including distant enhancers and 3D chromatin loops that bring them into promoter proximity. Unlike earlier models focused on chromatin marks, AlphaGenome jointly predicts chromatin accessibility, histone modifications, gene expression, and 3D contact maps—a comprehensive ‘genomic state’ for any sequence. For variant effect prediction, models like AlphaGenome compute the differential between predicted genomic states for reference and alternative alleles.

With these DL-based, megabase-scale models representing the ultimate frontier of all-encompassing learning, the interpretability of their learned representations is an active and fundamental area of research, yet still in its pioneering stage. Saliency maps have been developed to highlight input nucleotides contributing to predictions, and in silico saturation mutagenesis systematically tests the effect of every possible mutation at each position. These methods reveal that GLMs learn recognizable motifs (TF binding sites, splice signals) in early layers and combinatorial logic in deeper layers [95, 96]. However, some learned features do not map to known biology, and they may capture genuine but undocumented patterns, as well as spurious correlations such as GC content biases in training data [97]. Effort in this direction is paramount since validating that learned representations align with biology is essential before clinical deployment. Despite this, shifting to relying on black-box models in clinical practice is dangerous without appropriate validations. Sequence-based deep learning demonstrates that explicit feature engineering may be unnecessary when sufficient training data exists, but it raises interpretability challenges that hand-crafted features did not face.

### Protein language models

Just as GLMs learn from genomic sequences, protein language models (PLMs) represent the analogous development for amino-acid sequences, both driven by the same transition from manual feature engineering to latent space learning. PLMs learn predictive representations of protein sequences directly from evolutionary data, implicitly capturing conservation, structure and function without explicit feature specification. Most recently, these models have been largely based on the highly successful transformer architecture, the one underlying Large Language Models [98]. PLMs can thus be understood as a convergent representation of multiple coding feature categories described earlier: the training corpus of existing protein sequences implicitly encodes evolutionary constraint—amino acids that impair fitness are depleted, mirroring the conservation features; attention mechanisms capture co-evolutionary patterns that reflect structural contacts, paralleling the structure features; and population-level selection encoded in sequence databases mirrors the signal captured by population frequency features. The ESM (Evolutionary Scale Modeling) family from Meta AI exemplifies this approach. ESM-1b, a 650-million-parameter transformer trained on tens of millions protein sequences, uses masked language modeling to learn implicit representations of evolutionary constraint, protein structure, and functional domains [90]. For variant effect prediction, PLMs compute the log-likelihood ratio between mutant and wild-type amino acids, with more negative scores indicating predicted deleteriousness. This ‘zero-shot’ approach—requiring no training on pathogenicity labels—performs competitively with supervised methods on several benchmarks. Related models include ESM-1v (optimized for variant effect prediction from single sequences) [91], ESM-2 (improved architecture with better scaling properties) [99], and ProtT5 (based on the T5 encoder-decoder architecture from the Rost Lab) [100]. These models operate on single protein sequences; evolutionary signal emerges from the training corpus rather than explicit multiple sequence alignments (MSAs). Models requiring MSAs as input, such as MSA Transformer [101], achieve higher accuracy on average but at greater computational cost, though single-sequence PLMs can outperform MSA-based methods on protein families with shallow alignments where MSA signal is sparse.

In parallel with sequence-based PLMs, the AlphaFold architecture has been adapted for pathogenicity prediction in two distinct ways. PrimateAI-3D pioneered using AlphaFold-predicted structures as direct input to a 3D convolutional neural network, learning spatial patterns predictive of pathogenicity without manually defining structural features [45]. While not technically a PLM—since it operates on structural coordinates rather than sequence—PrimateAI-3D exemplifies the broader shift toward learning representations from foundation model outputs rather than hand-crafted features. AlphaMissense took instead a different approach: rather than using AlphaFold predictions as input features, it adapted the AlphaFold architecture itself for pathogenicity prediction [89]. By fine-tuning AlphaFold’s structural module on variant labels while incorporating evolutionary constraints from multiple sequence alignments, AlphaMissense learns which substitutions violate the structural and functional grammar encoded across homologous sequences. The release of pre-computed predictions for all ~71 million possible human missense variants, classifying 89% as either likely benign or likely pathogenic, provided the first proteome-wide pathogenicity landscape.

These diverse DL-based approaches are increasingly integrated by downstream tools. CADD v1.7 incorporates ESM-1v scores for missense variants [30]. VESPA uses ProtT5 embeddings to predict conservation without requiring sequence alignments [102]. VESPAg extends this approach for faster proteome-wide prediction [103]. The EVE model combines variational autoencoders with evolutionary information from multiple sequence alignments [104]. CPT-1 (Cross-Protein Transfer) demonstrates the power of feature integration by combining EVE scores, ESM-1v embeddings, and AlphaFold-derived structural features in an ensemble of logistic regression models trained on only five deep mutational scanning datasets [105]. Despite this minimal training data, CPT-1 achieves state-of-the-art performance, with 92.8% pairwise win rate against 97 VEPs on deep mutational scanning (DMS) benchmarks [16] (devoted to SNVs and short indels), illustrating that carefully selected features from complementary foundation models can outperform larger but less targeted approaches.

As for GLMs, understanding what PLMs learn is an open question addressed by interpretability research. Attention analysis of transformer-based PLMs reveals that specific attention heads encode biological structure: up to 63% of high-confidence attention weights connect amino acids that are spatially proximal in 3D structure (versus 1.3% expected by chance), and up to 51% of attention targets functional binding sites (versus 4.8% background) [106]. Moreover, individual attention heads have been shown to specialize: one attention head in TAPE [107], e.g. focuses 64% of attention on post-translational modification sites (versus 0.8% background) [106] Attention patterns also correlate with BLOSUM62 substitution matrices (r = 0.60–0.75), indicating that PLMs learn amino acid exchangeability patterns consistent with evolutionary observations [106]. An important caveat: attention weights are correlative, not necessarily causal explanations of model behaviour. Correlation with structural contacts demonstrates that PLMs learn structurally relevant representations, but does not prove that the model mechanistically relies on structure for its predictions. Still, more recent work using sparse autoencoders (SAEs) has also revealed that PLMs store biological concepts in ‘superposition’—individual neurons are polysemantic, encoding multiple concepts simultaneously. SAEs decompose these compressed representations into interpretable features: analysis of ESM-2 extracted 2309 features with strong biological concept alignment, compared to only 46 interpretable features from raw neuron activations. These features correspond to 143 distinct biological concepts including binding sites, catalytic residues, zinc finger domains, disulfide bonds, and disordered regions, demonstrating that PLMs learn explicit biological annotations implicitly from sequence alone [108]. Despite these advances, it has to be kept in mind that PLMs may also learn biases from their training data: sequences in UniProt have survived natural selection, potentially biasing predictions against severe loss-of-function variants while missing gain-of-function effects that depend on cellular context. The models may also struggle with protein–protein interaction disruptions and tissue-specific effects not captured by sequence alone.

## Methodological considerations

### Feature orthogonality

The proliferation of features and component scores raises fundamental questions about information independence. Highly correlated features capture redundant signal, while truly orthogonal features provide independent evidence. PLMs provide a striking example of this: ProtT5 embeddings predict sequence conservation with MCC = 0.596, compared to MCC = 0.608 for MSA-based methods—a difference of only 0.012 [102]. This near-equivalent predictive performance suggests that PLMs learn evolutionary constraint information comparable to explicit MSA-based conservation methods, though whether identical underlying representations are captured remains unclear. In an apparent parallel, the multiple conservation scores commonly used (phyloP, phastCons, GERP++, SiPhy) capture conceptually overlapping signal, as each measures constraint across a phylogeny. However, important methodological differences in how these scores are computed (e.g. single-site scoring versus HMM-based element detection) may still lead to only moderate empirical correlation between specific score pairs: the degree of predictive ability overlap cannot be inferred simply from the conceptual overlap. This distinction between conceptual similarity and empirical correlation is important, and shall be kept in mind: features measuring the same underlying biology through different methodologies can still provide complementary signal. Ultimately, while systematic quantification of feature independence across the enormous number of existing VEP tools against uniform benchmarks remains an open research question, practitioners should recognize that high correlation between component scores does not invalidate their joint use, particularly within non-linear modeling frameworks.

### Data circularity and benchmarking

Data circularity in VEP benchmarking has been formally characterized into two types [16, 109]. Type 1 circularity (variant-level) occurs when the exact variant used in evaluation was present in training. Type 2 circularity (gene-level) occurs when different variants from the same gene appear in both training and test sets—the model learns ‘gene X is pathogenic’ rather than evaluating variant-specific predictions. ‘Post-2014’ tools, proposed after the release of ClinVar, could be affected by circularity issue; the underlying issue is whether training and evaluation data overlap, which depends on specific tool implementations rather than publication year (Fig. 4).

**Figure 4:**
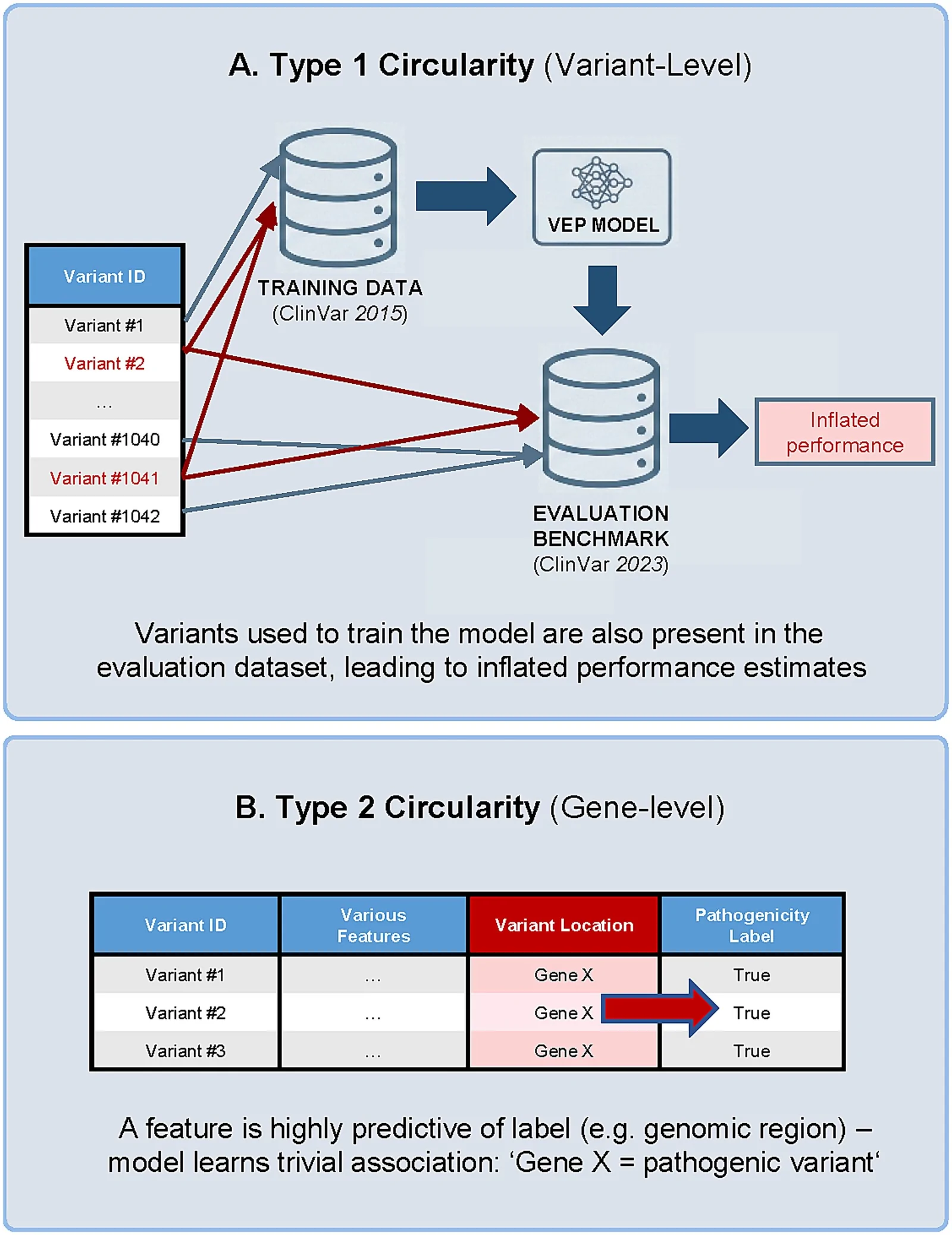
Two types of data circularity in VEP benchmarking. (A) Type 1 circularity (variant-level) occurs when variants present in the training data also appear in the evaluation benchmark, inflating performance estimates. (B) Type 2 circularity (gene-level) occurs when a feature such as genomic location is highly predictive of the pathogenicity label, causing the model to learn trivial associations rather than variant-specific biological signals.

A solution for Type 2 leakage is represented by cluster splitting, which consists of partitioning proteins into groups based on sequence identity (e.g. placing all sequences with >50% similarity into the same fold) and assigning entire groups exclusively to either training or testing sets. This strategy forces models to generalize to unseen protein families, representing the gold standard for ML validation in biology. Grimm et al. demonstrated that predictors confounded by gene-level circularity showed severe performance drops when evaluated on datasets curated to remove gene overlap, and that meta-predictors can appear superior to their component tools primarily due to compounded circularity [109]. Consistent with this, independent benchmarks on data entirely outside VEP training sets—including DMS experiments [15, 16] and population cohorts [110]—have consistently found that population-free methods—including protein language models and evolutionary generative models—and population-tuned methods rank among the top performers, suggesting that tools avoiding circularity generalize more robustly.

### Training data biases

Beyond circularity, systematic biases in training databases affect all VEPs (Fig. 2). ClinVar, the most widespread training data source for VEP tools, over-represents severe, early-onset Mendelian disease (ascertainment bias), genes with established disease associations (gene-level bias), missense variants over synonymous or non-coding (variant type bias) [111], and European ancestry populations (population bias) [112, 113]. These biases propagate to trained models, potentially limiting generalization. For example, benchmarking across 14 global ancestry groups has demonstrated that clinical-trained VEPs systematically over-predict pathogenicity in non-European populations, whereas population-free methods remain robust [114].

## Discussion and conclusion

The evolution of feature engineering in variant effect prediction reveals a clear trajectory: early tools relied on hand-crafted features encoding domain knowledge; modern tools increasingly compress biological context into learned latent spaces, where neural networks discover predictive patterns without explicit feature specification. Protein language models for coding variants represent the largest methodological advance in VEP since the introduction of ensemble predictors, which became widely integrated into clinical variant interpretation workflows. In parallel, AlphaFold derived structural information provides a complementary improvement beyond evolutionary patterns.

The outstanding performance of these modern tools, however, might not reflect superior learning algorithms per se, but rather more comprehensive integration of complementary biological signals—raising the question of how much performance gain stems from architecture versus input richness. With regard to practical deployment, annotation platforms and classical machine learning predictors are highly scalable and readily integrated into genomic analysis pipelines, often leveraging precomputed scores for rapid genome-wide annotation. Although deep learning and foundation models generally require greater computational resources, improvements in model optimization, GPU acceleration, and the availability of precomputed predictions are shifting the primary barriers to adoption from computational scalability toward software usability, interoperability, and integration into standardized clinical and research workflows.

Beyond accuracy and scalability, while these DL-based tools now outperform traditional methods, clinical guidelines continue to emphasize feature transparency.

The next generation of tools must bridge this gap—moving toward feature attribution that explains why a model predicts pathogenicity, even for DL-based approaches. While each methodological era has so far expanded the space of predictable variants, the current challenge is ensuring that expanded capability translates into clinical utility.

**Key Points**

- Early tools relied on explicit domain knowledge such as conservation scores and (for coding variants) physicochemical properties; modern language models compress this information into latent embeddings. Understanding this trajectory is essential to evaluate what has been gained—and what may have been lost—in interpretability and biological grounding.
- As genomic and protein language models replace hand-crafted features, understanding whether these models rediscover known biology or capture novel patterns remains critical for scientific insight and clinical trust.
- Addressing calibration heterogeneity, population biases in training data, and the gap between research benchmarks and real-world clinical utility remains an open challenge.
- Benchmarking standards are needed to eliminate data circularity.

## Supplementary Material

Bulloni_SuppTable1_bbag552

## Data Availability

There are no new data associated with this article.
